# Feasibility of Multimodal Artificial Intelligence Using GPT-4 Vision for the Classification of Middle Ear Disease: Qualitative Study and Validation

**DOI:** 10.2196/58342

**Published:** 2024-05-31

**Authors:** Masao Noda, Hidekane Yoshimura, Takuya Okubo, Ryota Koshu, Yuki Uchiyama, Akihiro Nomura, Makoto Ito, Yutaka Takumi

**Affiliations:** 1 Department of Otolaryngology, Head and Neck Surgery Jichi Medical University Shimotsuke Japan; 2 Department of Otolaryngology - Head and Neck Surgery Shinshu University Matsumoto Japan; 3 College of Transdisciplinary Sciences for Innovation Kanazawa University Kanazawa Japan

**Keywords:** artificial intelligence, deep learning, machine learning, generative AI, generative, tympanic membrane, middle ear disease, GPT4-Vision, otolaryngology, ears, ear, tympanic, vision, GPT, GPT4V, otoscopic, image, images, imaging, diagnosis, diagnoses, diagnostic, diagnostics, otitis, mobile phone

## Abstract

**Background:**

The integration of artificial intelligence (AI), particularly deep learning models, has transformed the landscape of medical technology, especially in the field of diagnosis using imaging and physiological data. In otolaryngology, AI has shown promise in image classification for middle ear diseases. However, existing models often lack patient-specific data and clinical context, limiting their universal applicability. The emergence of GPT-4 Vision (GPT-4V) has enabled a multimodal diagnostic approach, integrating language processing with image analysis.

**Objective:**

In this study, we investigated the effectiveness of GPT-4V in diagnosing middle ear diseases by integrating patient-specific data with otoscopic images of the tympanic membrane.

**Methods:**

The design of this study was divided into two phases: (1) establishing a model with appropriate prompts and (2) validating the ability of the optimal prompt model to classify images. In total, 305 otoscopic images of 4 middle ear diseases (acute otitis media, middle ear cholesteatoma, chronic otitis media, and otitis media with effusion) were obtained from patients who visited Shinshu University or Jichi Medical University between April 2010 and December 2023. The optimized GPT-4V settings were established using prompts and patients’ data, and the model created with the optimal prompt was used to verify the diagnostic accuracy of GPT-4V on 190 images. To compare the diagnostic accuracy of GPT-4V with that of physicians, 30 clinicians completed a web-based questionnaire consisting of 190 images.

**Results:**

The multimodal AI approach achieved an accuracy of 82.1%, which is superior to that of certified pediatricians at 70.6%, but trailing behind that of otolaryngologists at more than 95%. The model’s disease-specific accuracy rates were 89.2% for acute otitis media, 76.5% for chronic otitis media, 79.3% for middle ear cholesteatoma, and 85.7% for otitis media with effusion, which highlights the need for disease-specific optimization. Comparisons with physicians revealed promising results, suggesting the potential of GPT-4V to augment clinical decision-making.

**Conclusions:**

Despite its advantages, challenges such as data privacy and ethical considerations must be addressed. Overall, this study underscores the potential of multimodal AI for enhancing diagnostic accuracy and improving patient care in otolaryngology. Further research is warranted to optimize and validate this approach in diverse clinical settings.

## Introduction

The emergence of artificial intelligence (AI) has altered the landscape of medical technology, particularly in diagnosis, which leverages the identification of features based on imaging and physiological data [1-3]. In the field of otolaryngology, AI and deep learning models are being used for imaging; ongoing efforts focus on classifying diseases based on tympanic membrane images of middle ear disease [4-6]. Technological advancements, including deep learning and transfer learning using pretrained models, have resulted in an accuracy range of 70%-90% in models for analyzing otoscopic images [7]. There have also been advancements in its application, such as implementing smartphone-based point-of-care diagnostics [8]. However, these models rely on trained image data, require large image data sets, and do not consider patient information or clinical context. Consequently, the universality of these models is limited, and their optimal application in clinical practice remains unclear.

Recently, large-scale language-processing models have become available for general use. Further, 1 such model, the GPT-4, has demonstrated specialist-level medical knowledge through its language-processing abilities [9-11]. Since October 2023, GPT-4 Vision (GPT-4V) has gained the ability to evaluate image data, enabling a multimodal diagnostic approach that incorporates both language processing and image analysis [12]. GPT-4V enables the integration of patient information analysis and image-based deep learning models, providing valuable support in diagnosis and treatment, similar to decisions made in a clinical setting [13]. Multimodal AI, which bases diagnosis on multiple pieces of information, has been reported to be more effective than methods that rely on a single type of information. This is demonstrated in various medical applications, including the combination of pathology images with genomic information [14] and their use in liver cancer [15] and cervical cancer [16], where imaging information is integrated. In otorhinolaryngology, there have been few reports; however, efforts to incorporate AI for otoscopic images could further improve the quality of care.

In this study, we aimed to investigate the effectiveness of a multimodal approach using GPT-4V to diagnose middle ear disease. This approach was designed to integrate patient-specific data (age, sex, and chief complaint) with tympanic membrane images to assess the accuracy of the versatile GPT-4V. The model’s accuracy was compared with physicians’ diagnoses to validate its effectiveness in image-based deep learning. The potential future development of the multimodal AI approach for classifying middle ear diseases is also discussed.

## Methods

### Study Design

**Figure 1:**
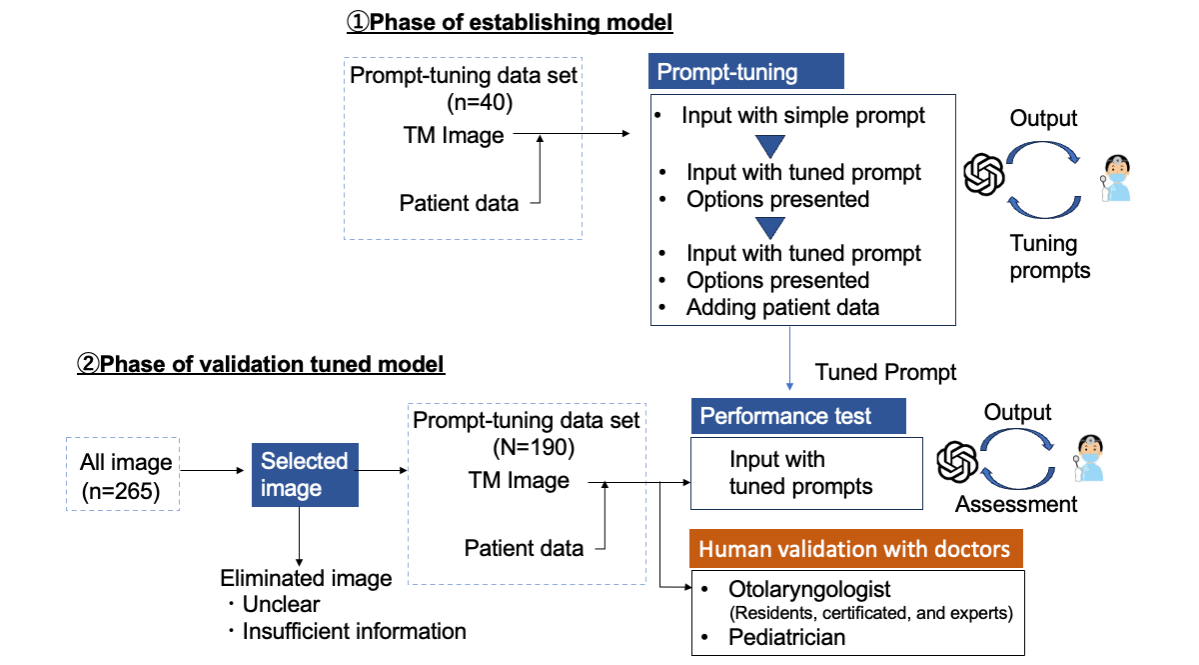
Overview of this study. The model was divided into two phases: (1) establishment and (2) tuned model validation.TM: tympanic membrane.

GPT-4V has been available as an image recognition model since September 25, 2023. This study’s design was divided into two phases: (1) establishing a model with appropriate prompts and (2) validating the ability of the optimal prompt model to classify images (Figure 1).

### Correct Otoscopic Images and Patient Information

This study included 305 otoscopic images of middle ear disease obtained from patients who visited Shinshu University or Jichi Medical University between April 2010 and December 2023. The endoscope used was an Olympus ENF-VH and ENF-V3 (Olympus), and the video system was an Olympus VISERA ELITE OTV-S190. Further, 1 image was obtained from each patient. We excluded images with poor quality and those in which multiple diseases were suspected. The remaining images were classified into 4 disease categories: acute otitis media (AOM), middle ear cholesteatoma (chole), chronic otitis media (COM), and otitis media with effusion (OME). The final diagnoses were based on the judgment of the otolaryngologists who treated the patients. These images were accompanied by patient-specific information, such as age, sex, and chief complaint (eg, fever, otalgia, otorrhea, ear fullness, deafness, facial palsy, dizziness, and tinnitus). We excluded images taken after otologic surgery. Of note, only 1 image was obtained from each patient.

### GPT-4V Settings and Prompt Tuning

The GPT-4V settings were established using prompts reported in previous studies [17,18]. Briefly, conditions and prompts for providing answers were verified using 10 images for each disease. According to a report on prompts [19], image data or patient information were manually input into GPT-4V, and the generated results were evaluated by the physicians (MN and HY).

### Accuracy Verification of GPT-4V Using the Optimal Prompt Model

The model with the optimal prompt created was used to verify the diagnostic accuracy of GPT-4V on 190 images (37 in AOM, 53 in chole [6 in congenital, 47 in acquired], 51 in COM, and 49 in OME), which were different from those for tuning prompts. To account for the variability in responses, each administration was performed 3 times, and responses that were answered 2 or more times were considered to be the actual response.

### Comparison of AI Accuracy With Physician Accuracy

To compare the diagnostic accuracy of GPT-4V with that of physicians, 30 clinicians completed a web-based questionnaire consisting of 190 images.

The web-based survey included tympanic membrane images and patient information (age, sex, and chief complaint) in a 4-choice question format. The respondents included 8 certificated pediatricians, 8 otolaryngology residents, 8 certificated otolaryngologists, and 6 experts in otolaryngology (more than 15 years of experience).

To show the trend in the percentage of correct responses according to the difficulty of the questions, the questions were divided into 3 levels (easy, normal, and hard) according to the overall percentage of correct responses by physicians, and the percentage of correct responses for each level and each question was compared between the GPT-4V and all doctors, otolaryngologists, and pediatricians.

### Ethical Considerations

Patient information was anonymized to protect privacy and used only with the approval of the Ethics Committee of the Shinshu University School of Medicine (6088).

### Statistical Analysis

Groups were compared by 1-way ANOVA. Subsequently, multiple comparison tests (the Bonferroni method) were used to compare groups. Statistical significance was set at *P*<.05. A 1-sample proportion test was used to compare the performance of the physician with that of GPT-4V in terms of the correct response rate.

## Results

### Establishment of Optimal Prompts

In the initial stage, we sought an optimal input method using 10 images for each disease (AOM, chole, COM, or OME; 40 images total). First, we input only images or options; GPT mostly requires clinical information, such as patient history and symptoms, although no response regarding the disease was generated (Figure 1 and Multimedia Appendix 1). Second, the names of the 4 diseases were added as candidate answers, but again, no response regarding the disease was generated. When detailed patient information, such as age, sex, and main symptoms, was inputted, GPT-4V provided answers, indicating that input images with patient data were the optimal prompt for testing the accuracy of GPT-4V.

### Accuracy Validation of the Multimodal AI Approach

**Figure 2:**
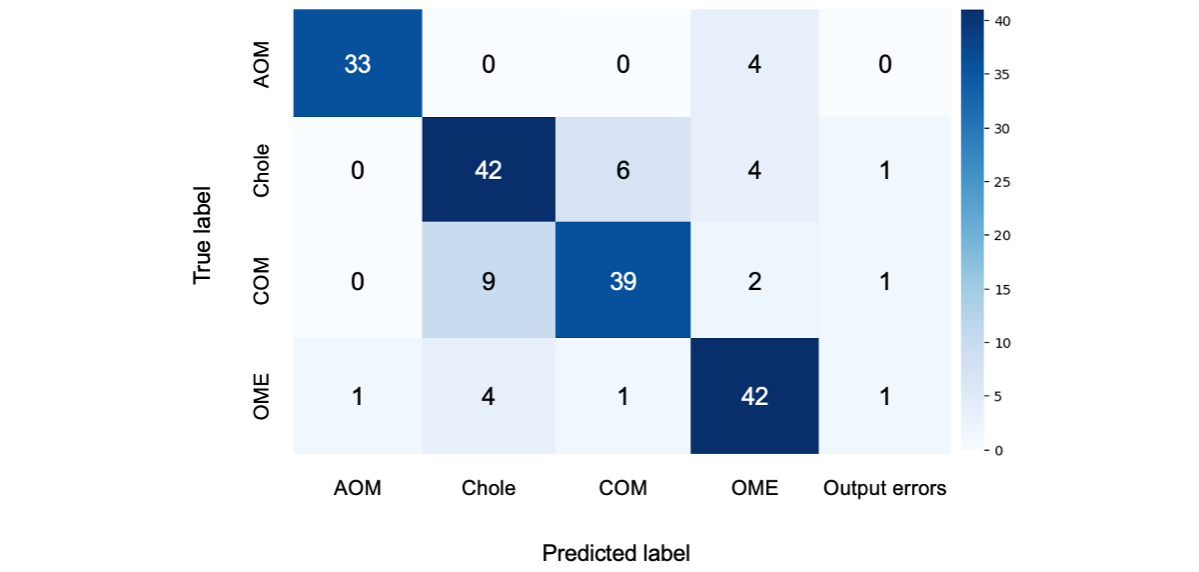
Confusion matrix of GPT-4V for classifying 4 middle ear diseases. AOM: acute otitis media; chole: middle ear cholesteatoma; COM: chronic otitis media; GPT-4V: GPT-4 Vision; OME: otitis media with effusion.

The performance of the multimodal AI approach in this study for classifying middle ear diseases was validated, with an overall diagnostic accuracy of 82.1% for the GPT-4V-based analysis. Disease-specific accuracy rates were 89.2% for AOM (true positives [TP]=33, false positives [FP]=1, false negatives [FN]=4, precision=0.97, recall=0.89, *F*_1_-score=0.93), 76.5% for COM (TP=39, FP=7, FN=12, precision=0.85, recall=0.76, *F*_1_-score=0.8), 79.3% for cholesteatoma (TP=42, FP=13, FN=11, precision=0.76, recall=0.79, *F*_1_-score=0.78), and 85.7% for OME (TP=42, FP=10, FN=7, precision=0.81, recall=0.86, *F*_1_-score=0.83; Figure 2).

**Figure 3:**
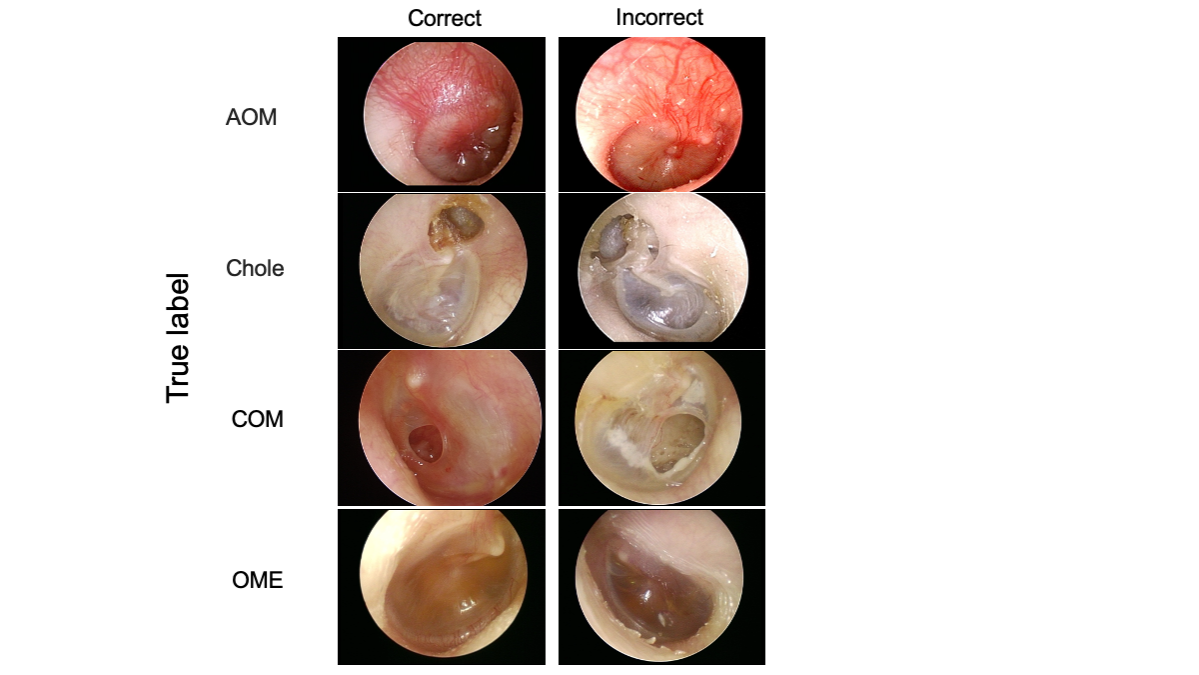
Representative images of correct and incorrect GPT-4V classifications for 4 middle ear diseases. The left side shows the correct images for GPT-4V classification, and the right side shows the incorrect images for GPT-4V. AOM: acute otitis media; chole: middle ear cholesteatoma; COM: chronic otitis media; GPT-4V: GPT-4 Vision; OME: otitis media with effusion.

These results indicate high discrimination among various disease types; however, there were also some incorrect responses. Representative images of correct and incorrect GPT-4V classifications for each disease are shown in Figure 3.

### Comparison of Diagnostic Accuracy by Physicians and GPT-4V

**Figure 4:**
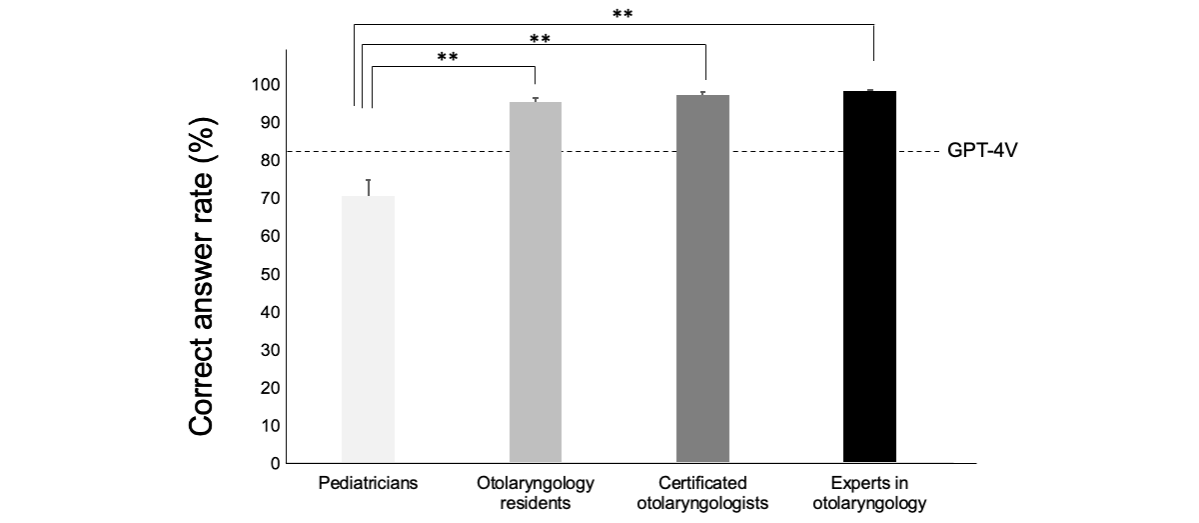
Result of human validations with doctors of TM images and patients’ data. The graph shows the average correct rate for doctors (pediatricians, otolaryngology residents, certificated otolaryngologists, and experts in otolaryngology), and the dotted line shows the correct answer rate of GPT-4V. GPT-4V: GPT-4 Vision; TM: tympanic membrane. **P value <.01.

The same images with patients’ information used by GPT-4V were evaluated by pediatricians (n=8), otolaryngology residents (n=8), certificated otolaryngologists (n=8), and experts in otolaryngology (n=6), and the diagnostic accuracy of each group was compared. The mean diagnostic accuracy was 70.6% (SE 4.2%) for pediatricians, 95.5% (SE 1%) for otolaryngology residents, 97.3% (SE 0.8%) for certificated otolaryngologists, and 98.2% (SE 0.4%) for experts in otolaryngology. ANOVA revealed significant differences among the 4 groups (*F*_1_=13.43, *P*<.001). In the post hoc comparison, a significant difference was observed between pediatricians and the other 3 groups (*P*<.001). The GPT-4V correct response rate was 82.1%, surpassing that of pediatricians by 11.5% and trailing behind otolaryngologists by an average of just over 10% (Figure 4).

The accuracy rates for specific diseases were as follows: 92.3% for AOM (pediatricians 80.4%, otolaryngology residents 94.9%, certificated otolaryngologists 97%, and experts in otolaryngology 98.2%), 95.9% for COM (pediatricians 89.5%, otolaryngology residents 96.6%, certificated otolaryngologists 99.8%, and experts in otolaryngology 98.4%), 81.8% for chole (pediatricians 46%, otolaryngology residents 93.2%, certificated otolaryngologists 93.6%, and experts in otolaryngology 98.4%), and 93.7% for OME (pediatricians 81.6%, otolaryngology residents 97.2%, certificated otolaryngologists 99%, and experts in otolaryngology 98%).

**Figure 5:**
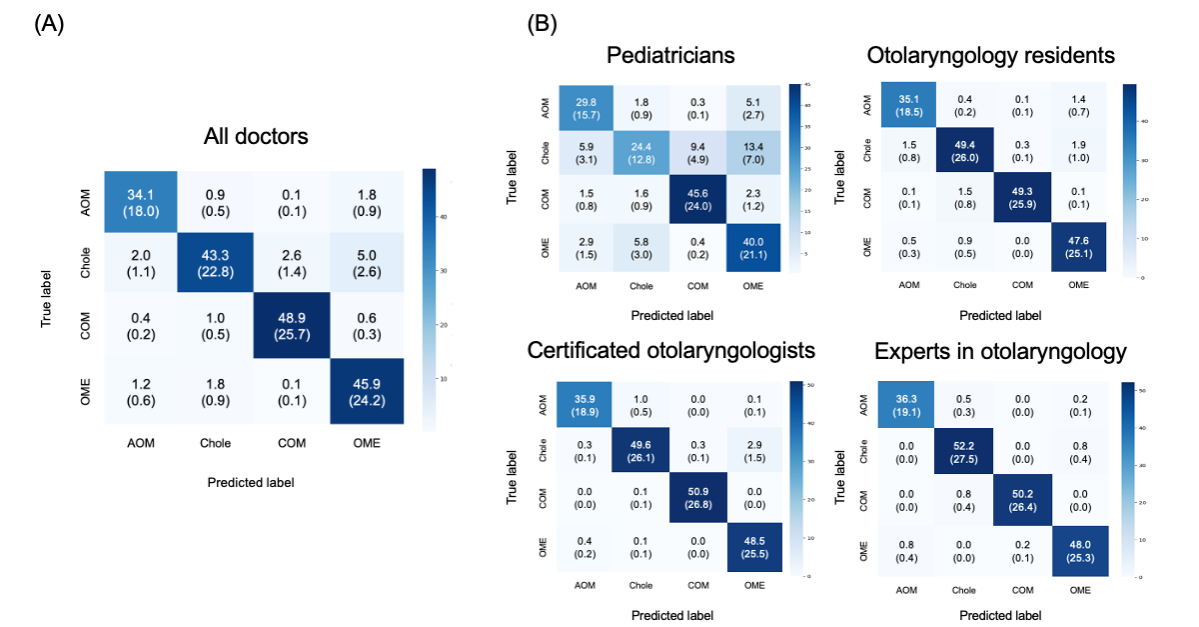
Confusion matrix of doctors (pediatricians, otolaryngology residents, certificated otolaryngologists, and experts in otolaryngology) for classifying 4 middle ear diseases. (A) Confusion matrix of all doctors (N=30). The average (percentage of total responses) is shown. (B) Confusion matrix of doctors in each group: pediatricians (n=8), otolaryngology residents (n=8), certificated otolaryngologists (n=8), and experts in otolaryngology (n=6). The averages of each group (percentage of total responses) are shown. AOM: acute otitis media; chole: cholesteatoma; COM: chronic otitis media; OME: otitis media with effusion.

In the confusion matrix of all doctors, there was a notable tendency to misclassify chole as OME and AOM as OME. Among pediatricians, there were more errors in classifying chole as AOM or COM (Figure 5).

Regarding the difference in the trend of the percentage of correct answers between GPT-4V and physicians according to the difficulty of the questions, even the percentage of correct answers for GPT-4V tended to decrease gradually from 85.7% for easy, 84% for normal, and 71.1% for hard questions (Table 1).

Furthermore, compared with otolaryngologists, GPT-4V had a significantly lower percentage of correct answers for all questions (99.7% for easy, 97.1% for normal, and 90.8% for hard questions; all *P*<.001). In contrast, the results of the “hard” and “normal” groups were similar. Compared with pediatricians, the GPT-4V outperformed the pediatricians in easy questions with 96.6%, although no statistically significant difference was observed (*P*=.006). However, the GPT-4V had a predominantly higher percentage of correct answers for normal (76.3%, *P*=.07) and hard questions (45.4%, *P*<.001).

**Table 1:** Comparison of the scores by GPT-4 Vision (GPT-4V) and human validation with physicians across various difficulty levels (N=190).

Difficulty level	Questions, n (%)	GPT-4V (mean %)	All doctors				Otolaryngologists				Pediatricians		
			Mean % (95% CI)	Differences	*P* value	Mean % (95% CI)		Differences	*P* value	Mean % (95% CI)		Differences	*P* value
Easy (>95%)	77 (40.5)	85.7	97.8 (97.4-98.2)	12.1	<.001^a^	99.7 (99.5-99.9)		14.0	<.001^a^	96.6 (95.3-97.9)		10.9	.006^a^
Normal (>85%, <95%)	75 (39.5)	84	90.4 (89.7-91.0)	6.4	.13	97.1 (96.2-98.0)		13.1	<.001^a^	76.3 (73.6-79.0)		–7.7	.07
Hard (<85%)	38 (20)	71.1	76.8 (73.7-79.8)	5.7	.44	90.8 (87.2-94.3)		19.7	<.001^a^	45.4 (39.5-51.3)		–25.7	<.001^a^

^a^Statistically significant.

## Discussion

### Principal Results

In this study, we assessed the accuracy of the GPT-4V multimodal AI approach in classifying middle ear disorders, yielding the following three key findings. First, GPT-4V, a general-purpose model focusing on large-scale language models, achieved approximately 80% accuracy in classifying middle ear disease. The model’s performance, evaluated using images and patient data, was superior to that of nonotolaryngologists, although it was lower than the average accuracy of otolaryngologists. Second, the GPT-4V was able to classify diseases when patient information and disease options were input. Further improvements in accuracy could be achieved with more detailed patient information. Third, accuracy varied by disease, suggesting the potential for optimizing AI usage and improving accuracy by understanding the specificity of GPT-4V in classifying particular diseases.

### Comparison With Prior Work

The GPT-4V model has undergone training and uses 0-shot learning, which recognizes image features based on natural language to classify diseases based on image information and previously learned disease features [20]. GPT-4V can yield effective results with fewer resources than previous deep learning models, which typically require a large amount of image data, computational resources, time, and parameter adjustments for training. By inputting new information rather than simply classifying image data, it becomes possible to tailor diagnoses and diagnostic aids for each individual. Furthermore, GPT-4V and other large-scale language processing models feature prompt development that is appropriate for its usage purposes, since the accuracy of such models varies depending on the prompt adjustments.

Compared with physicians’ accuracy, the model’s performance in this study was higher than that of a pediatrician but lower than that of an otolaryngologist. In a previous comparison between deep learning and humans, Crowson et al [21] classified 22 tympanic membrane images and found that the deep learning model achieved an accuracy of 95.5%, compared with an accuracy of 65% for 39 clinicians. Suresh et al [22] also reported that a machine-learning model created from 1000 images was more effective than pediatricians, with an accuracy rate of 90.6%, surpassing the clinicians’ accuracy of 59.4%. Our results indicated that the model did not reach the proficiency level of otolaryngologists; however, it could be valuable for using tympanic membrane images in medical practice outside of otolaryngology. In particular, GPT-4V judgments predominantly exceeded pediatricians' correct response rates for questions with normal to hard difficulty, suggesting that the present model may be useful for nonotolaryngologists who have difficulty in making such judgments.

Moreover, previous reports on deep learning classification models have determined the presence or absence of inflammation and exudates based on photographs alone. Further studies are needed to identify the optimal stage in the examination for implementing the image classification model and the subsequent policy decisions that should follow.

GPT-4V allows for the classification of diseases using patient information. While comments about medical or harmful content (with restrictions on medical advice) may result in a lower correct response rate, informative or educational responses are still possible if they are well-informed. Efforts have been made to use large language models (LLMs) to improve the accuracy of prompts. Therefore, it is possible to develop appropriate prompts for medical imaging and middle ear disorders. The accuracy of the LLM is expected to further improve with the development of prompts that are specifically tailored for medical imaging and middle ear disease [23,24].

For the clinical application of the GPT-4V model, collecting clinical data and adjusting parameters are needed to further improve its diagnostic accuracy for each middle ear disease. Upon reviewing the incorrect responses of GPT-4V for each disease, we found that chole might demonstrate a retraction pocket, which may be mistaken for a perforation. However, images with keratin debris accumulation in the retraction pocket were less prone to misclassification. In cases of COM with calcification, a white lesion was considered to be chole calcification, emphasizing the importance of distinguishing between these 2 diseases. AOM cases without the chief complaint of acute inflammation (fever, ear pain, or ear discharge) were occasionally misclassified, even with characteristic findings such as a bulging tympanic membrane, suggesting that GPT-4V was likely to prioritize patients' information over images. In OME cases, a white lesion was sometimes considered to be a pearly tumor (chole) or tympanic membrane perforation (COM), particularly when it involved a small amount of effusion or air. For physicians, chole and AOM were often misidentified as other diseases and OME, respectively. When comparing the GPT-4V model with the entire group of physicians, the percentage of correct responses was generally higher among the physicians. However, the GPT-4V diagnostic accuracy for chole was higher than that of pediatricians, indicating that GPT-4V could help nonotolaryngologists diagnose chole. In a previous report, a dedicated AI model had a diagnostic accuracy of approximately 90% for chole [25]; therefore, the combination of such a system and GPT-4V would be useful to improve the accuracy of chole detection.

As demonstrated in this study, the application of AI, including LLM, is believed to offer advantages in terms of improving efficiency and providing assistance in clinical work, enabling the delivery of high-quality medical care, and overcoming language barriers in medicine. The use of GPT-4V has already been reported to diagnose complicated cases [26], and its application can be expanded by integrating it with imaging information. In the field of orthopedics, trials are underway to determine treatment methods based on MRI reports [27], showcasing the effectiveness of GPT-4V as an aid in image interpretation. GPT has been shown to return answers and provide details about the disease, including risk factors and treatment methods. This allows for the evaluation of images alone and assists in medical treatment. Such insights are valuable for understanding the practical use and challenges of AI in real-world applications. Unlike the simplistic deep learning models of the past, the LLM can enhance accuracy by presenting evidence for judgments and asking a series of questions. When used by physicians with a certain level of specialized knowledge, the LLM effectively aids judgment, leading to increased efficiency in medical care. GPT-4V provides answers in just a few seconds, which is significantly shorter than the time it takes a physician to provide a diagnosis, thereby confirming its efficiency. GPT-4V can be used on smartphones, potentially making medical treatment more location-independent. However, there are associated risks, including the reliance on AI for medical care, misdiagnoses due to system malfunctions, and patient information leakage. ChatGPT (OpenAI, Microsoft Corporation) is trained based on information up to a certain period but may respond differently at different times or provide answers using outdated criteria. Furthermore, legal and personal literacy measures must be developed to protect personal information and address ethical concerns. Foreign countries and the United Nations are actively promoting laws and regulations governing the use of AI [28,29].

### Limitations

In total, one limitation of this study is the use of a limited number of images (N=190). Further analysis is required to assess the impact of using a larger data set that encompasses various diseases. Additionally, as there are large variations in the quality of otoscopic images, accurate diagnosis might be challenging in some cases.

The recognition and content of the answers may change depending on the doctor, clinics, and designed prompt; the accuracy may also change due to changes in the image quality used or the method used to capture the image. While this is common to deep learning, the advantage of GPT, which does not require prior training, is that it is not affected by the data to be trained; thus, the possibility of such changes is considered to be small.

For these reasons, further exploration is needed on strategies for handling challenging images and facilitating open-ended responses without giving predefined options. Furthermore, because of the rapid pace of technological evolution, it is essential to regularly fine-tune and make a standalone model that ensures reliability and consistency over time.

### Conclusions

A multimodal AI approach using GPT-4V has revealed a potential new diagnostic approach for classifying middle ear diseases. This confirms the ability of AI to assist in clinical diagnosis and identify disease-specific features. The significant improvement in accuracy compared with conventional deep learning models indicates that even general-purpose AI technology can assist in medical treatment with a certain level of accuracy. It can be applied to highly specialized diagnoses, depending on the method. Further improvements in diagnostic accuracy are expected in future studies by integrating more diverse data types.

## Appendix

Multimedia Appendix 1 Representative image and prompt of this study. (A) Representative image of input and output to GPT-4 Vision. Input can be combined with text and images in input to obtain output. (B) Example of changing the prompt content and an output that asks for patient information. By presenting a concept as ORDER and adding conditions as restriction, appropriate prompts were attempted to be developed. In the output, it is required to input patient information such as age, medical history, and chief complaint. (C) An example of an answer with an optimized prompt. Present the diagnosis, the rationale for the diagnosis, and treatment and prevention methods.

## Abbreviations

AI: artificial intelligence
AOM: acute otitis media
chole: middle ear cholesteatoma
COM: chronic otitis media
FN: false negative
FP: false positive
GPT-4V: GPT-4 Vision
LLM: large language model
OME: otitis media with effusion
TP: true positive
