# Correction: Feasibility of Multimodal Artificial Intelligence Using GPT-4 Vision for the Classification of Middle Ear Disease: Qualitative Study and Validation

**DOI:** 10.2196/62990

**Published:** 2024-07-09

**Authors:** Masao Noda, Hidekane Yoshimura, Takuya Okubo, Ryota Koshu, Yuki Uchiyama, Akihiro Nomura, Makoto Ito, Yutaka Takumi

**Affiliations:** 1 Department of Otolaryngology, Head and Neck Surgery Jichi Medical University Shimotsuke Japan; 2 Department of Otolaryngology - Head and Neck Surgery Shinshu University Matsumoto Japan; 3 College of Transdisciplinary Sciences for Innovation Kanazawa University Kanazawa Japan

In “Feasibility of Multimodal Artificial Intelligence Using GPT-4 Vision for the Classification of Middle Ear Disease: Qualitative Study and Validation” (JMIR AI 2024;3:e58342) the authors noted one error.

In the original author, no equal contributors were specified. This has been changed as follows:

Masao Noda*, Hidekane Yoshimura*

*These authors contributed equally

The correction will appear in the online version of the paper on the JMIR Publications website on July 9, 2024, together with the publication of this correction notice. Because this was made after submission to PubMed, PubMed Central, and other full-text repositories, the corrected article has also been resubmitted to those repositories.

