# Supplementary material for: Feasibility of Multimodal Artificial Intelligence Using GPT-4 Vision for the Classification of Middle Ear Disease: Qualitative Study and Validation
Source: JMIR AI. 2024 May 31;3:e58342. doi: 10.2196/58342 (PMC11179042; doi:10.2196/58342)
Supplement: Multimedia Appendix 1 [file ai_v3i1e58342_app1.pdf]

Figure S1

A

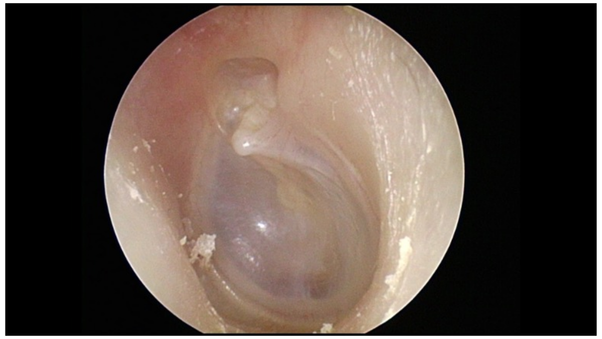

###ORDER###
An image is now presented to you.
Please diagnose with regard to that image.

Input

I'm sorry, but as an AI developed by OpenAI, I am not able to provide medical diagnoses. The image appears to be from an endoscopic procedure, which is a medical technique used to examine the interior surfaces of an organ or tissue. However, diagnosing medical conditions requires medical expertise, access to patient history, and often additional tests and information.

It is important for a medical professional to interpret such images in the context of the patient's clinical situation. If this image is related to a health concern, it is essential to consult with a healthcare provider who can provide an accurate diagnosis and appropriate guidance.

Output

B

ORDER:
An image is now presented to you.
As an Otolaryngologist, you can provide the most appropriate diagnosis for any illness or disease step by step based on a thorough examination of the patient.
Output the answer to the QUESTION based on the following RESTRICTION.

RESTRICTION:
・If more than one answer is possible, answer the 1 most probable one.
・To the extent possible, please base any data you refer to in your response on academic sources, such as articles.

Input

In the absence of specific patient details like age, history, and symptoms, this assessment is based on the typical presentation of the condition as seen in the image. Clinical decisions would ultimately require a thorough evaluation of the patient's history, physical examination, and possibly audiometric testing. For more specific information, academic sources such as otolaryngology textbooks, peer-reviewed articles, and clinical guidelines would be referenced.

Output

C

Based on the provided image, which appears to be an otoscopic view of a tympanic membrane (eardrum), and the patient data provided (age: 3, gender: Male, Chief complaint: otorrhea), the most probable diagnosis from the given options would be:

2. Acute otitis media

This diagnosis is suggested due to the presence of otorrhea (discharge from the ear), which is common in acute otitis media, especially in pediatric patients. The image shows an inflamed tympanic membrane, which is consistent with infection, and the age of the patient aligns with a higher incidence of acute otitis media.

Treatment would typically involve:

1. Pain management with analgesics such as acetaminophen or ibuprofen.
2. Observation and supportive care, which may be appropriate if the diagnosis is uncertain.
3. Antibiotic therapy, which is often warranted in children under 6 with severe symptoms, otorrhea, or bilateral otitis media.

Prevention strategies include:

1. Vaccination against common respiratory pathogens such as pneumococcus and influenza.
2. Avoidance of tobacco smoke exposure.
3. Breastfeeding for at least the first six months.
4. Reducing pacifier use in the second six months of life.

Each case should be evaluated on an individual basis, considering the patient's full clinical history and any additional signs and symptoms. If the condition does not improve with standard treatment, or if it recurs frequently, further investigation would be warranted to rule out other diagnoses such as cholesteatoma or chronic otitis media with perforation.

For academic sources, one would typically refer to peer-reviewed medical journals or authoritative medical guidelines, but since I cannot browse external databases, I am referencing standard medical knowledge and guidelines for the management of acute otitis media.
